# Risk factors of necrotizing enterocolitis in very low birth weight infants: a meta-analysis

**DOI:** 10.3389/fped.2026.1750560

**Published:** 2026-02-06

**Authors:** Liming Bao, Jinyan Huang

**Affiliations:** The Second Affiliated Hospital of Wenzhou Medical University, Zhejiang, China

**Keywords:** infants, meta-analysis, necrotizing enterocolitis, risk factors, very low birth weight

## Abstract

**Background:**

This study aims to explore the primary risk factors for necrotizing enterocolitis (NEC) in very low birth weight (VLBW) infants through meta-analysis, providing scientific evidence for clinical prevention and treatment.

**Methods:**

A systematic search was conducted in PubMed, Embase, Cochrane Library, and Web of Science databases for studies investigating risk factors for NEC in VLBW infants, covering the period from database inception to October 10, 2025. Eligible studies included case-control studies, cohort studies, and cross-sectional studies meeting the inclusion criteria. Quality assessment was performed using the NOS and AHRQ scores. Data were pooled using Stata 15 software with a random-effects model.

**Results:**

a total of 16 research articles involving 179,289 patients included, meta-analysis results suggest that Small Gestational Age [OR = 1.35, 95% CI (1.14, 1.60)], red blood cells transfusion [OR = 1.75, 95% CI (1.26, 2.43)], maternal hypertensive disorders [OR = 1.27, 95% CI (1.03, 1.57)], patent ductus arteriosus [OR = 1.56, 95% CI (1.30, 1.88)], sepsis [OR = 1.87, 95% CI (1.22, 2.87)] were associated with NEC in very low birth weight infants.

**Conclusion:**

This systematic meta-analysis consolidates and confirms previously reported associations between several clinical factors and the risk of necrotizing enterocolitis in very low birth weight infants. The findings support the association of small gestational age, red blood cell transfusion, maternal hypertensive disorders, patent ductus arteriosus, and sepsis with an increased risk of NEC.

**Systematic Review Registration:**

PROSPERO CRD420251149565.

## Background

Necrotizing enterocolitis (NEC) is one of the most severe gastrointestinal diseases in the neonatal period, characterized by intestinal inflammation, ischemic necrosis, and compromised barrier function (1, 2). It is often accompanied by systemic inflammatory response and may even lead to multiple organ failure (3). NEC predominantly affects preterm infants, particularly those with very low birth weight (VLBW, birth weight <1,500 g), exhibiting a significantly higher incidence than term newborns (4). While advances in neonatal intensive care have markedly improved survival rates among VLBW infants, NEC remains a primary contributor to persistently high morbidity and mortality in this population (5). Research indicates that following NEC onset, VLBW infants not only face high acute mortality risks but may also develop long-term sequelae such as chronic intestinal dysfunction, neurodevelopmental delay, and growth restriction, imposing a heavy burden on both patients and families (6).

The pathogenesis of NEC is complex, involving multiple interacting factors. Existing research indicates that immature intestinal barrier development, dysbiosis of the gut microbiota, intestinal ischemia and hypoxia, abnormal immune responses, and nutritional factors are all closely associated with the onset of NEC (7). VLBW infants, due to their underdeveloped intestines, exhibit significantly reduced digestive absorption capacity and barrier defense capabilities, making them susceptible to external stimuli that trigger inflammatory responses (8). Furthermore, mechanical ventilation, parenteral nutrition, and broad-spectrum antibiotics are often required for preterm infants (9). While these interventions improve survival rates, they may also increase the risk of gut microbiota disruption and intestinal mucosal damage, further elevating NEC incidence (10).

Epidemiological data indicate that the incidence of NEC in VLBW infants ranges from approximately 5%–10%, rising to over 20% in extremely preterm infants (gestational age <28 weeks) (11). Mortality rates increase significantly with decreasing birth weight and gestational age. For infants weighing <1,000 g, NEC-related mortality may exceed 30% (12). The severity of NEC is reflected not only in high mortality but also in its potential to trigger complications such as intestinal perforation, peritonitis, and multiple organ failure. These complications complicate clinical management, prolong hospital stays, and increase medical costs and financial burdens on families (13).

Although previous meta-analyses have explored risk factors for NEC in children and neonates, limited sample sizes, substantial differences in study designs, and inconsistencies in risk factor assessment metrics and analytical methods across studies (14, 15) have hindered the development of unified clinical intervention strategies (16). Consequently, this study focuses specifically on VLBW infants to identify high-risk factors for NEC in this population. This is crucial for guiding early clinical interventions, optimizing feeding management, reducing disease incidence, and improving patient outcomes.

## Methods

This systematic evaluation and meta-analysis will strictly follow the PRISMA (Preferred Reporting Items for Systematic Reviews and Meta-Analyses) guidelines (17). And it is registered in Prospero with registration number CRD420251149565.

### Literature retrieval

A systematic search was conducted in PubMed, Embase, Web of Science, and the Cochrane Library databases from their inception to October 1, 2025, to identify literature related to risk factors for NEC in VLBW infants. The search strategy combined Medical Subject Headings (MeSH) terms and free-text keywords, with primary search terms including: “necrotizing enterocolitis”, “very low birth weight”, “risk factor”. The detailed search strategy is provided in Supplementary Table S1. To minimize omissions, reference lists of included studies and citations from relevant reviews were manually searched.

### Inclusion criteria

Study Type: Prospective or retrospective observational studies (cohort studies, case-control studies, cross-sectional studies).
Study Population: very low birth weight infants.
Exposure: Clearly reported incidence of necrotizing enterocolitis
Study Results: Provided or calculable effect sizes for risk factors (OR values, RR values, and their 95% confidence intervals).
Assessable literature quality with complete data.

### Exclusion criteria

Studies with duplicate publications or overlapping data.
Case reports, conference abstracts, reviews, commentaries, or animal studies.
Studies failing to clearly distinguish necrotizing enterocolitis or lacking extractable data.
Studies where full-text access is unavailable.

### Study selection

During the literature screening process, two researchers independently used EndNote 21 software to initially screen the literature obtained from the search, first through the titles and abstracts, and then to exclude literature that clearly did not meet the inclusion criteria. Subsequently, the remaining literature was reviewed by reading the full text in its entirety to further determine whether it met the inclusion and exclusion criteria. In case of disagreement between the two researchers during the screening process, it would be resolved through discussion and negotiation; if the negotiation still failed to reach a consensus, a third researcher would be invited to adjudicate to ensure the objectivity and consistency of the screening process.

### Data extractions

This study was conducted by two researchers who independently extracted relevant data from the eligible literature using an Excel sheet based on the inclusion criteria. The extraction included the basic information of the study (first author, year of publication, country and study design), the basic characteristics of the study population [sample size, number of NEC, gender, gestational age (week) and birth weight(g)], diagnosis of NEC and the statistical model used in the regression analysis. In the process of data extraction, if two investigators disagreed on the data, it would be resolved through negotiation, and if no agreement could be reached, a third investigator would adjudicate to ensure the accuracy and consistency of data extraction.

### Quality evaluation

The types of studies included in this study will be assessed using different quality assessment tools: for case-control and cohort studies, the NOS (Newcastle-Ottawa Scale) (18) quality assessment tool will be used, which evaluates the intrinsic bias of the studies through three main domains (study selectivity, comparability, and assessment of outcomes), focusing on sample selection, the relationship between exposure and relationship between exposure and outcome, and control of confounders; these quality assessment tools ensure that the included studies have a high-quality evidence base. For cross-sectional studies, this study will use the AHRQ quality (19) assessment tool to evaluate quality. This tool primarily assesses the rationality of the study design, the representativeness of the sample selection, the clarity of the definitions of exposure and outcome, the accuracy of the data collection process, the rationality of the statistical methods, and the completeness of the report.

### Statistical analysis

In this study, the random effects model was adopted due to the high degree of heterogeneity among the included studies. The risk ratio (OR) and corresponding 95% confidence interval (CI) were extracted for each study and then pooled together. To account for the variability between studies, the random effects model was chosen, which provides a more generalized estimate of the overall effect size when there is significant heterogeneity. The heterogeneity of the combined studies was assessed using the I² statistic. If the I² value was greater than 50%, it was indicative of substantial heterogeneity, and further exploration of potential sources of this heterogeneity was required. In cases of high heterogeneity, sensitivity analyses may be performed to identify any factors that could influence the overall effect sizes. To detect publication bias, a funnel plot was generated, and its asymmetry was examined. If the funnel plot showed signs of asymmetry, Egger's test was performed to assess the statistical significance of the bias. A *p*-value of <0.05 suggests the presence of publication bias, while a *p*-value of >0.05 indicates no significant bias. If necessary, the trim-and-fill method may be applied to adjust for any potential publication bias and verify the robustness of the results. Finally, the combined effect sizes will be reported as odds ratios (ORs) with their respective 95% confidence intervals (CIs) to facilitate the interpretation of the findings and allow for statistical inference.

## Results

### Literature retrieval results

**Figure 1:**
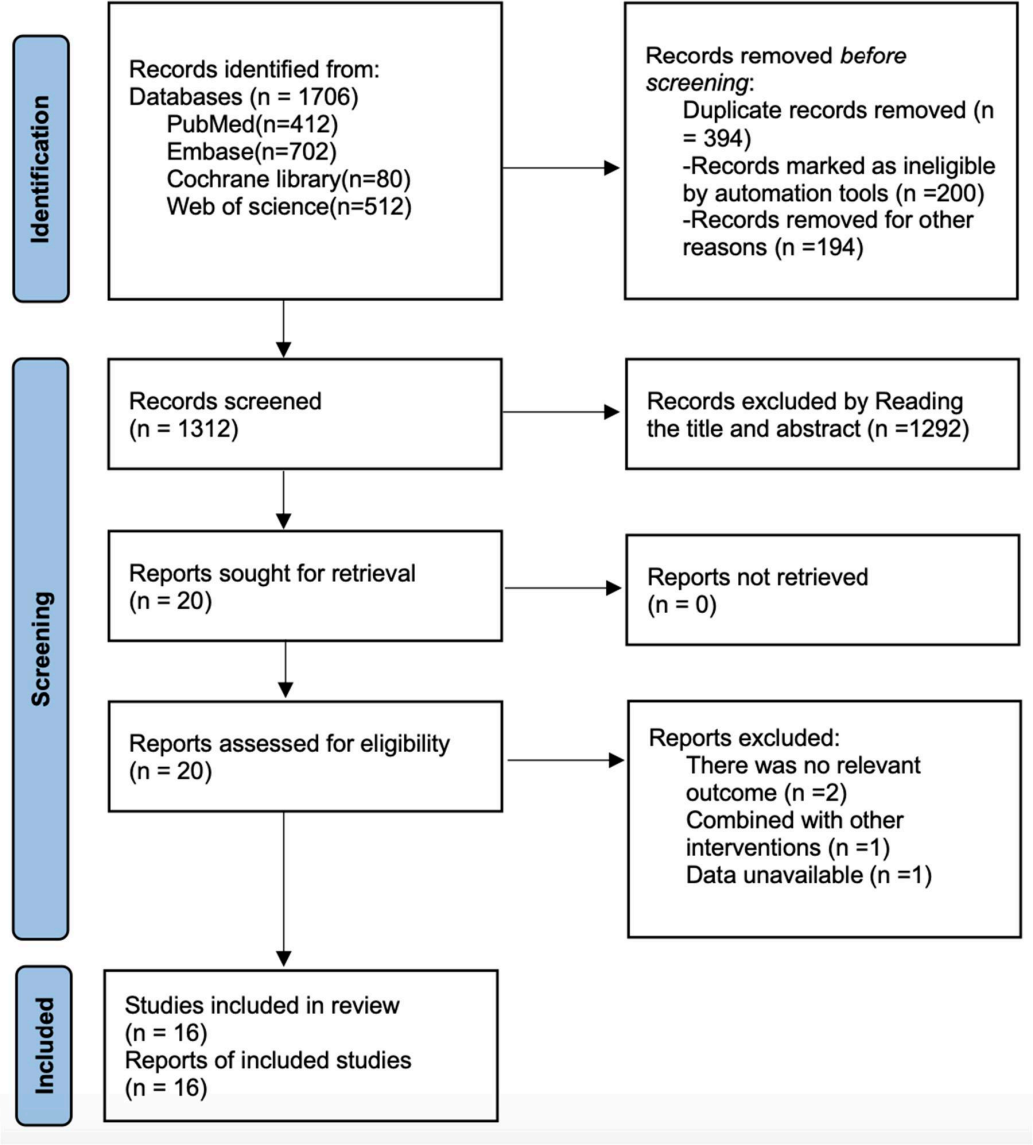
Literature search nnnflow chart.

As shown in Figure 1, a total of 1,706 articles were retrieved from PubMed (*n* = 412), Embase (*n* = 702), Cochrane Library (*n* = 80), and Web of Science (*n* = 512). After removing 394 duplicate records, 1,292 articles were excluded based on title and abstract screening, and 4 articles were excluded after full-text review. Ultimately, 16 articles (20–35) were included.

### Basic characteristics of included studies

This study included a total of 16 research articles involving 179,289 patients, comprising 5 case-control studies, 1 cross-sectional study, and 10 cohort studies. All patients had a birth weight <1,500 g, and NEC was confirmed by imaging findings of pneumatosis intestinalis and/or surgical specimens. Specific baseline characteristics are detailed in Table 1.

**Table 1:** Table of basic characteristics of literature.

Author	Year	Study design	Country	Sample size	No of NEC	Gender (M/F)	Gestational age (week)	Birth weight(g)	Diagnosis of NEC	Regression model	Risk factors
AlFaleh	2014	Case-control	Saudi Arabia	152	17	82/70	28	1,042	NR	Multivariable logistic regression	R2;
Bain	2014	Cohort study	USA	98,523	3,966	48,520/50003	29	<1,500	NR	Multivariable logistic regression	
Bashiri	2003	Cross sectional	Israel	211	17	140/71	28.5	<1,500	NEC was confirmed by imaging findings of pneumatosis intestinal and/or surgical specimens	Multivariable logistic regression	R1; R3; R4;
Bertino	2009	Case-control	Italy	34	17	17/17	29.2	1,065	NR	Multivariable logistic regression	R3;
Boo	2012	Cohort study	Malaysia	3,601	222	2,000/1,601	29	<1,500	NEC was confirmed by imaging findings of pneumatosis intestinal and/or surgical specimens	Multivariable logistic regression	R4; R5;
Chen	2020	Cohort study	China	461	23	261/200	30.7	<1,500	NEC was confirmed by imaging findings of pneumatosis intestinal and/or surgical specimens	Multivariable logistic regression	
Dollberg	2005	Cohort study	Israel	6,146	343	3,103/3,042	28.5	1,002	NEC was confirmed by imaging findings of pneumatosis intestinal and/or surgical specimens	Multivariable logistic regression	R4;
Feng	2003	Cohort study	China	292	39	173/119	28.5	1,087.64	NEC was confirmed by imaging findings of pneumatosis intestinal and/or surgical specimens	Multivariable logistic regression	R1;
Gitau	2023	Cohort study	Kenya	200	15	95/105	29	1,102	NEC was confirmed by imaging findings of pneumatosis intestinal and/or surgical specimens	Multivariable logistic regression	R1; R2; R5;
Kamanga	2025	Case-control	China	42,413	167	NR	31	1,310	NEC was confirmed by imaging findings of pneumatosis intestinal and/or surgical specimens	Multivariable logistic regression	R4;
Kim	2025	Cohort study	Korea	5,310	718	3,310/2,000	28	825	NEC was confirmed by imaging findings of pneumatosis intestinal and/or surgical specimens	Multivariable logistic regression	R1; R3;
Lee	2017	Cohort study	China	354	26	180/174	32.1	1,225	NEC was confirmed by imaging findings of pneumatosis intestinal and/or surgical specimens	Multivariable logistic regression	R1; R2; R5;
Martin	2013	Case-control	Spain	576	30	200/376	27.2	<1,500	NEC was confirmed by imaging findings of pneumatosis intestinal and/or surgical specimens	Multivariable logistic regression	R2;
Pansombat	2024	Case-control	Thailand	195	60	100/95	39.8	<1,500	NEC was confirmed by imaging findings of pneumatosis intestinal and/or surgical specimens	Multivariable logistic regression	R5
Patel	2016	Cohort study	USA	598	54	298/300	27.9	1,015	NEC was confirmed by imaging findings of pneumatosis intestinal and/or surgical specimens	Multivariable Cox Regression	R2;
Riskin	2021	Cohort study	Israel	20,223	1,439	10,223/10,000	28	<1,500	NEC was confirmed by imaging findings of pneumatosis intestinal and/or surgical specimens	Multivariable logistic regression	R1; R3; R4; R5

NR, not reported; NEC, necrotizing enterocolitis; R1, small gestational age; R2, red blood cells transfusion; R3, maternal hypertensive disorders; R4, patent ductus arteriosus; R5, sepsis.

### Risk bias results

As shown in Table 2, for cohort studies, 5 studies scored 9 points, 4 studies scored 8 points, and 1 study scored 7 points. For case-control studies, 2 studies scored 9 points, 1 study scored 8 points, and 2 studies scored 7 points. For cross-sectional studies, the research quality was moderate. The overall quality of the articles included in this study was high.

**Table 2:** Quality evaluation result.

Cross-sectional											
Study	Whether the source of the information is clear	Whether exposed and non-exposed groups are listed	Whether a time was given to identify patients	If not, population derived, whether the subjects were consecutive	Whether the subjective factors of the evaluator cover up other aspects of the research object	Any assessment performed to ensure quality is described	The rationale for excluding any patients from the analysis was explained	Describe measures to evaluate and/or control for confounding factors	Explain how missing data were handled in the analysis	Response rates and the completeness of data collection are summarized	If there is follow-up, identify the percentage of patients with expected incomplete data or follow-up results
Bashiri et al. (21)	Yes	Unclear	Yes	Yes	Yes	Yes	Yes	Yes	Yes	Unclear	Yes
Cohort study											
Study	Representativeness of the exposed group		Selection of non-exposed groups	Determination of exposure factors	Identification of outcome indicators not yet to be observed at study entry	Comparability of exposed and unexposed groups considered in design and statistical analysis	design and statistical analysis	Adequacy of the study's evaluation of the outcome		Adequacy of follow-up in exposed and unexposed groups	Total scores
Bain et al. (33)	*		*	*	*	**	*	*		*	9
Boo and Cheah et al. (34)	*		*	*	/	**	*	*		*	8
Chen et al. (23)	*		*	*	/	**	*	*		*	8
Dollberg et al. (24)	*		*	*	*	**	*	*		*	9
Feng et al. (25)	*		*	*	*	**	*	*		*	9
Gitauet al. (26)	*		*	*	*	**	*	*		*	9
Kim et al. (28)	*		*	*	/	**	/	*		*	7
Lee et al. (29)	*		*	*	*	**	*	*		*	9
Patel et al. (31)	*		*	*	/	**	*	*		*	8
Riskin et al. (32)	*		*	*	/	**	*	*		*	8
Case control											
Study	Is the case definition adequate?		Representativeness of the cases	Determination of control group	Definition of Controls	Comparability of cases and controls based on the design or analysis	Ascertainment of exposure	Same method of ascertainment for cases and controls		Non response	Total scores
AlFaleh et al. (20)	*		*	*	*	**	*	*		*	9
Bertino et al. (22)	*		*	*	/	**	/	*		*	7
Kamanga et al. (27)	*		*	*	*	**	*	*		*	9
Martin 2013	*		*	*	/	**	*	*		*	8
Pansombat et al. (35)	*		*	*	/	**	/	*		*	7

### Meta- analysis results

#### Small gestational age

**Figure 2:**
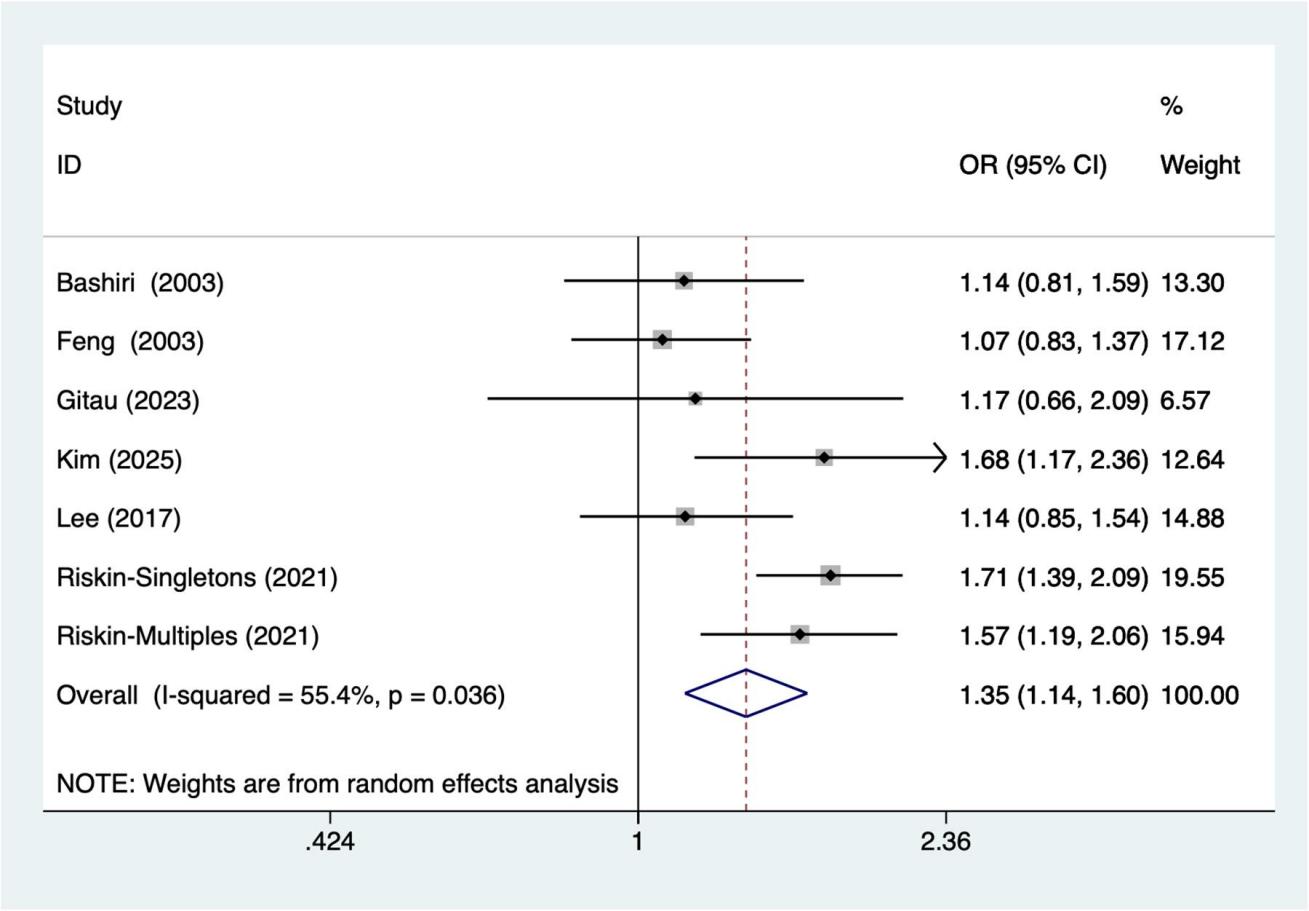
Forest plot of meta-analysis for small gestational Age.

7 studies reported Small Gestational Age. Heterogeneity testing (*I*^2^ = 55.4%, *P* = 0.036) was conducted using a random-effects model. The pooled analysis (Figure 2) showed that small gestational age was associated with a higher occurrence of NEC in very low birth weight infants [OR = 1.35, 95% CI (1.14, 1.60)]. Due to substantial heterogeneity, sensitivity analysis was conducted by sequentially excluding studies, results (Supplementary Figure S1) indicate stable findings unaffected by individual studies.

### Red blood cells transfusion

**Figure 3:**
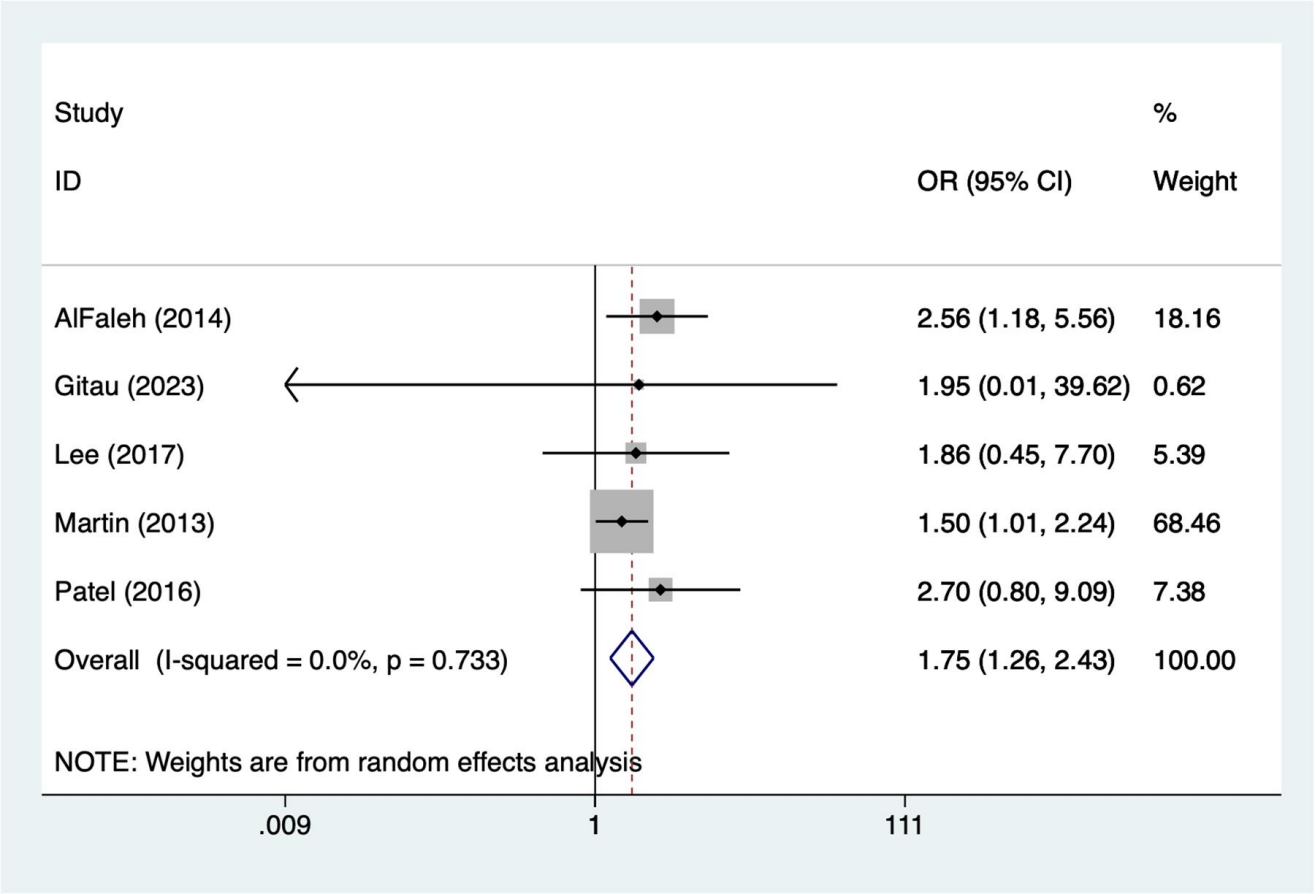
Forest plot of meta-analysis for red blood cells transfusion.

5 studies reported red blood cells transfusion. Heterogeneity testing (*I*^2^ = 0%, *P* = 0.733) was conducted using a random-effects model. The pooled analysis (Figure 3) indicated that red blood cell transfusion was associated with a higher occurrence of NEC in very low birth weight infants [OR = 1.75, 95% CI (1.26, 2.43)].

### Maternal hypertensive disorders

**Figure 4:**
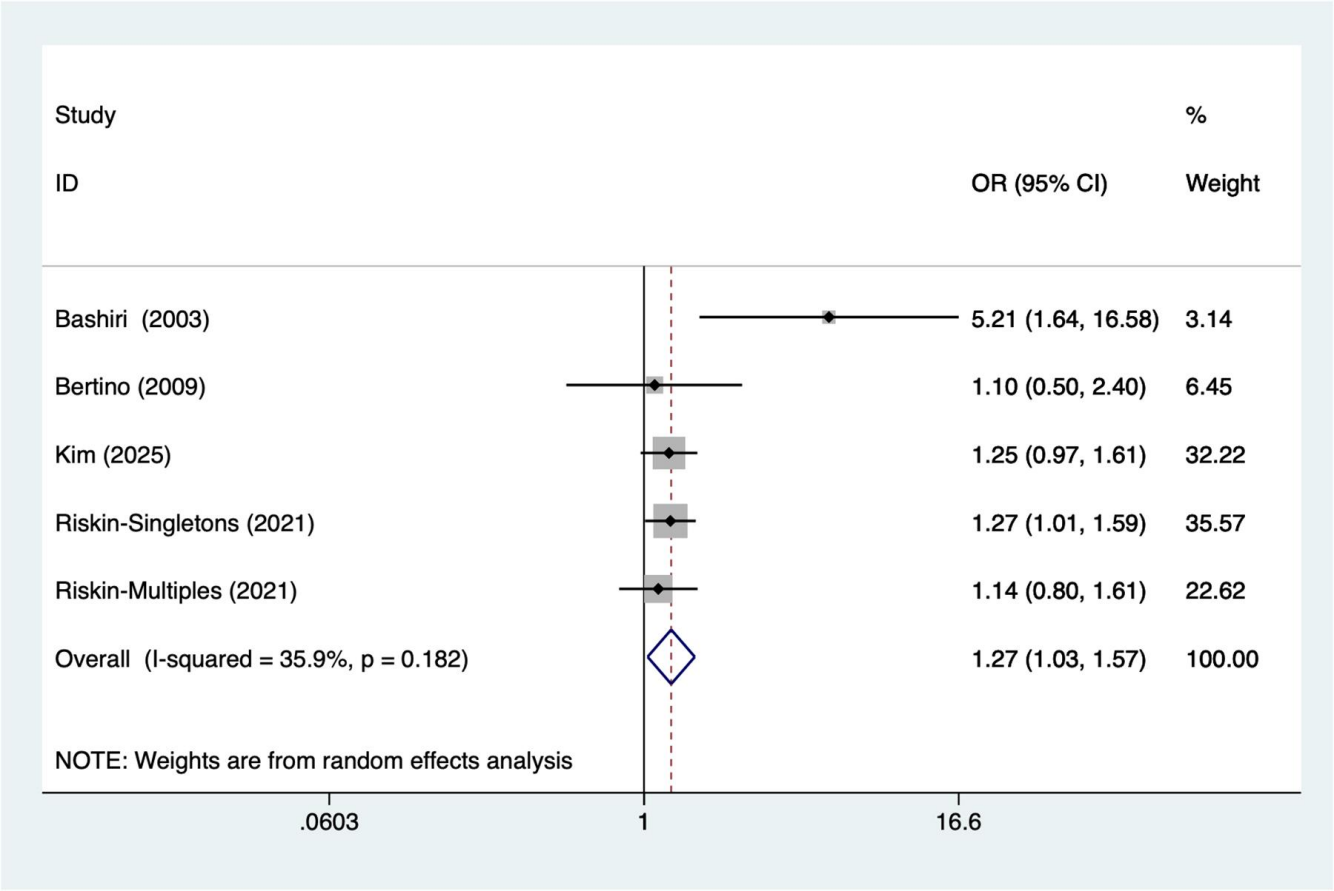
Forest plot of meta-analysis for maternal hypertensive disorders.

5 studies reported maternal hypertensive disorders. Heterogeneity testing (*I*^2^ = 35.9%, *P* = 0.182) was conducted using a random-effects model. The pooled analysis (Figure 4) showed that maternal hypertensive disorders were associated with NEC in very low birth weight infants [OR = 1.27, 95% CI (1.03, 1.57)].

### Patent ductus arteriosus

**Figure 5:**
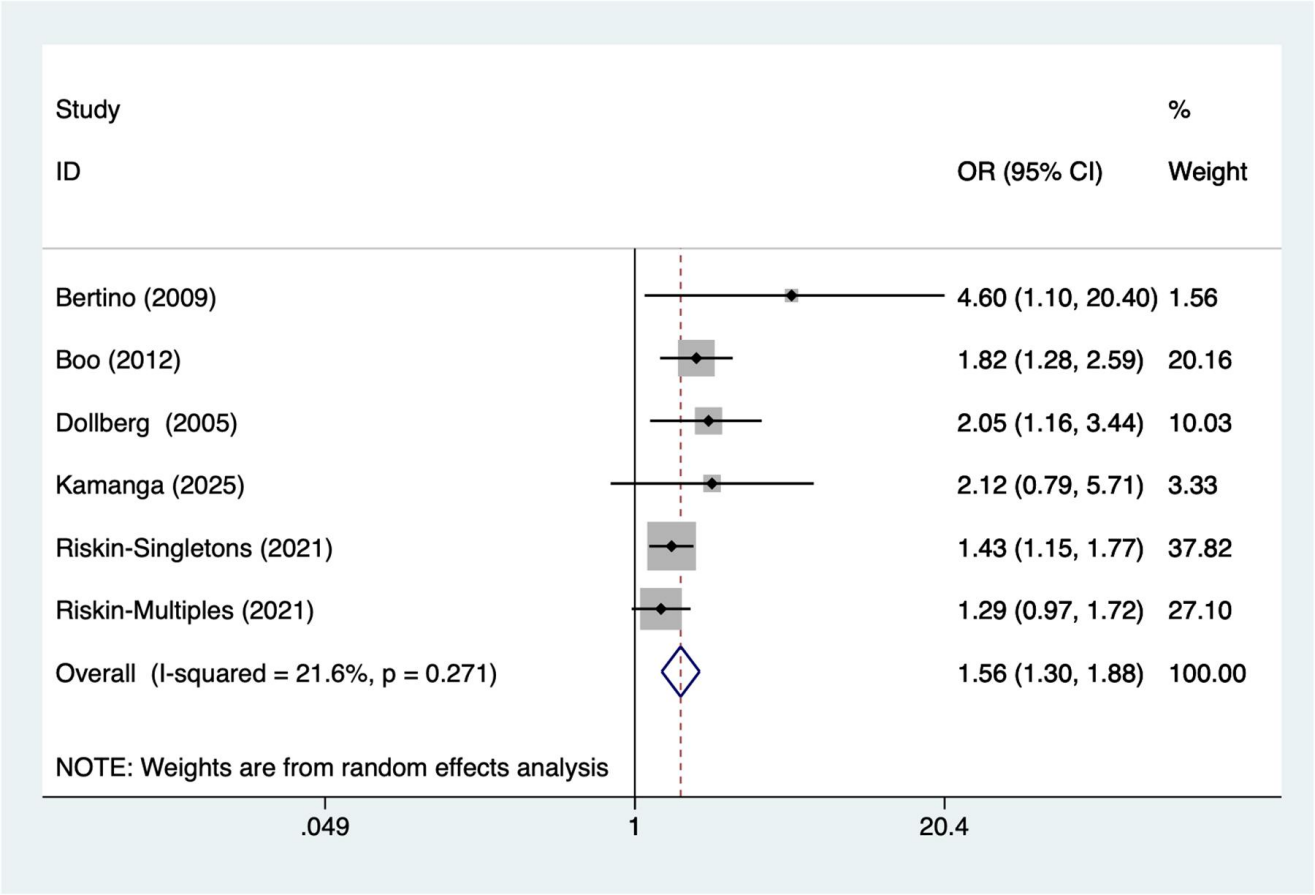
Forest plot of meta-analysis for patent ductus arteriosus.

6 studies reported patent ductus arteriosus. Heterogeneity testing (*I*^2^ = 21.6%, *P* = 0.271) was conducted using a random-effects model. The pooled analysis (Figure 5) showed that patent ductus arteriosus was associated with NEC in very low birth weight infants [OR = 1.56, 95% CI (1.30, 1.88)].

### Sepsis

**Figure 6:**
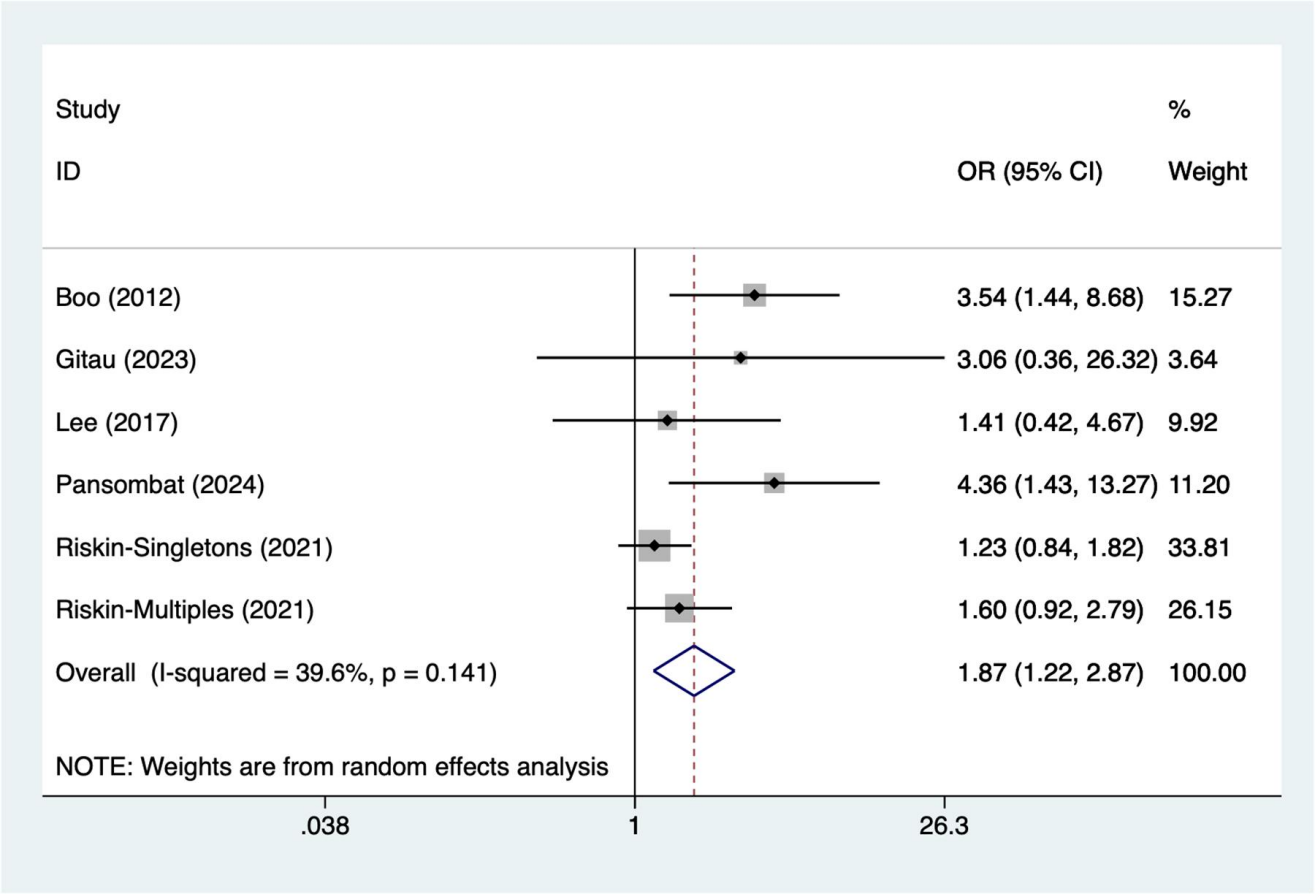
Forest plot of meta-analysis for sepsis.

6 studies reported sepsis. Heterogeneity testing (*I*^2^ = 39.6%, *P* = 0.141) was conducted using a random-effects model. The pooled analysis (Figure 6) indicated that sepsis was associated with NEC in very low birth weight infants [OR = 1.87, 95% CI (1.22, 2.87)].

### Publication bias

This study assessed publication bias using funnel plots and Egger's test. Funnel plot results (Supplementary Figures S2–S6) suggest a low likelihood of publication bias. Egger's test results showed no significant interaction for Small Gestational Age (*p* = 0.12), red blood cell transfusion (*p* = 0.223), maternal hypertensive disorders (*p* = 0.145), patent ductus arteriosus (*p* = 0.215), and sepsis (*p* = 0.831) suggest a low likelihood of publication bias.

## Discussion

In this meta-analysis, we systematically synthesized existing evidence to evaluate the associations between several previously reported factors and the risk of necrotizing enterocolitis in very low birth weight infants. The findings confirm that preterm birth, red blood cell transfusion, maternal history of hypertension, patent ductus arteriosus, and sepsis are associated with an increased risk of NEC. By providing a quantitative and comprehensive assessment of these associations, this study contributes to the current scientific knowledge base, offering a clearer clinical context for risk stratification, prevention strategies, and clinical management in VLBW infants.

The association between Small Gestational Age (SGA) and NEC risk in VLBW infants, identified in this study, aligns with numerous previous studies, all of which demonstrate a significant relationship between lower gestational age and the occurrence of NEC (36). Infants with SGA are at greater risk due to their underdeveloped immune systems and compromised intestinal barrier functions, which make them more susceptible to infection (37). This finding emphasizes the importance of early identification and intervention for these infants. Moreover, the compromised gut microbiota and intestinal function in preterm infants contribute to increased intestinal permeability, facilitating pathogen invasion and promoting NEC development (38, 39). This study strengthens the evidence supporting early nutritional and immune support as key interventions to reduce the incidence of NEC in SGA infants.

Red blood cell transfusion, another significant risk factor identified in our analysis, has been reported as a potential trigger for NEC (40). The association between transfusion and NEC occurrence is well-documented, with previous research indicating that red blood cell transfusion can induce oxidative stress and alter immune responses, leading to impaired intestinal function (41). In addition, the transfusion–associated reperfusion process may play a critical role in the pathogenesis of NEC. In VLBW infants with pre-existing intestinal hypoperfusion or anemia, red blood cell transfusion can lead to sudden restoration of blood flow, resulting in ischemia–reperfusion injury. This process promotes the generation of reactive oxygen species, endothelial dysfunction, and inflammatory cascades, which may further compromise the immature intestinal mucosa. Moreover, the quantity of red blood cells transfused may also influence NEC risk. Larger transfusion volumes or rapid correction of anemia may exacerbate intestinal reperfusion injury and increase circulatory instability, particularly in hemodynamically fragile VLBW infants, insufficient blood supply may lead to intestinal hypoxia following transfusion, further damaging the intestinal mucosa and increasing the risk of infection (42). This study provides further validation of these findings and underscores the need for careful consideration of transfusion practices, focusing on reducing unnecessary transfusions to mitigate the risk of NEC. Maternal hypertensive disorders, such as gestational hypertension and preeclampsia, also represent potential risk factors for NEC. Our analysis revealed a significant association between maternal hypertensive disorders and NEC occurrence in VLBW infants (43). Hypertensive states may impact fetal development through multiple pathways, leading to inadequate fetal blood supply and consequently increasing the risk of preterm birth (44). The pathophysiological mechanisms underlying this relationship involve impaired intestinal barrier development and heightened susceptibility to infection, both of which are known to promote NEC development in preterm infants. Our findings emphasize the importance of monitoring and managing maternal hypertensive conditions to reduce the risk of NEC in VLBW infants. Our analysis indicates that it may increase the risk of NEC. The altered blood flow direction caused by patent ductus arteriosus may affect intestinal perfusion in infants, leading to intestinal hypoxia and impaired blood flow, thereby promoting the development of NEC (45). This finding suggests that early diagnosis and treatment of patent ductus arteriosus are crucial for reducing the incidence of NEC. Appropriate cardiovascular monitoring and intervention in VLBW infants can improve intestinal blood supply and reduce NEC incidence. Sepsis, a common and severe infectious complication in VLBW infants, was found in this study to be significantly associated with NEC. Sepsis may damage the intestinal barrier through multiple pathways, including abnormal immune responses, dysbiosis of the gut microbiota, and exacerbated inflammatory reactions (46). In VLBW infants, the immature immune system makes infections more likely to trigger systemic inflammatory responses, which may precipitate NEC (47). Therefore, preventing and treating sepsis is a key strategy to reduce NEC incidence in VLBW infants. Enhanced infection prevention, early sepsis detection, and prompt treatment will help reduce the risk of NEC.

In this study, we conducted heterogeneity tests for various risk factors and found that some factors (small gestational age) exhibited high heterogeneity (*I*^2^ = 55.4%). This indicates that while differences exist among studies in terms of research design, patient characteristics, and interventions, the overall trend for these factors still suggests an association with NEC risk. To further validate the robustness of these findings, we conducted sensitivity analyses. The results showed that excluding individual studies sequentially did not significantly alter the outcomes, indicating that the conclusions of this study possess good stability and reliability.

### Strengths and limitations

This study's strengths lie in its use of systematic literature searches and meta-analysis methods, synthesizing results from multiple studies to provide reliable evidence on risk factors for NEC in VLBW infants. By incorporating seven studies on Small Gestational Age and five on red blood cell transfusion, among others, this research ensures data diversity and representativeness. Additionally, the use of a random effects models effectively accounted for heterogeneity among studies, and sensitivity analyses validated the robustness of the findings. These strengths confer high credibility and clinical guidance value to this study in identifying NEC risk factors for VLBW infants.

Although this study provides important evidence on risk factors for NEC in VLBW infants, several limitations exist. First, some included studies originated from a single country or region, potentially introducing geographical bias that limits the broad applicability of the findings. Second, variations in risk factor definitions and measurement criteria across studies may compromise consistency and comparability of findings. Although we adjusted for heterogeneity in our analysis, uncontrolled confounding factors may still exist. Furthermore, the predominantly retrospective nature of most studies introduces potential reporting and selection biases. Consequently, prospective and multicenter studies are warranted to further validate these findings.

### Clinical significance

The findings of this study hold significant clinical implications. First, understanding the risk factors for NEC in VLBW infants can assist clinicians in identifying high-risk infants and implementing personalized interventions to reduce NEC incidence. For instance, enhanced intestinal protection measures and immune support should be prioritized for Small Gestational Age infants; the necessity of blood transfusions should be carefully weighed for infants requiring red blood cell transfusions; and close monitoring with prompt treatment of infections is essential for infants at risk of sepsis. Additionally, maternal hypertension and conditions such as patent ductus arteriosus require thorough attention in both prenatal and postnatal management.

## Conclusion

This systematic meta-analysis consolidates and confirms previously reported associations between several clinical factors and the risk of necrotizing enterocolitis in very low birth weight infants. The findings support the association of small gestational age, red blood cell transfusion, maternal hypertensive disorders, patent ductus arteriosus, and sepsis with an increased risk of NEC. These associations are biologically plausible and may be mediated through disturbances in intestinal perfusion, immune regulation, and vulnerability to infection. Nevertheless, given the heterogeneity across included studies and the presence of potential bias, well-designed prospective multicenter studies are still required to further verify these associations and refine risk stratification in this population.

## Data Availability

The original contributions presented in the study are included in the article/Supplementary Material, further inquiries can be directed to the corresponding author.
