# Supplementary material for: Risk factors of necrotizing enterocolitis in very low birth weight infants: a meta-analysis
Source: Front Pediatr. 2026 Feb 6;14:1750560. doi: 10.3389/fped.2026.1750560 (PMC12920506; doi:10.3389/fped.2026.1750560)
Supplement: Supplementary file 1 [file Datasheet1.docx]

Table S1 detailed search strategy

((("Risk Factors"[Mesh]) OR (((((((((((((((((((Risk Factors[Title/Abstract]) OR (Factor, Risk[Title/Abstract])) OR (Risk Factor[Title/Abstract])) OR (Population at Risk[Title/Abstract])) OR (Populations at Risk[Title/Abstract])) OR (Risk Scores[Title/Abstract])) OR (Risk Score[Title/Abstract])) OR (Score, Risk[Title/Abstract])) OR (Risk Factor Scores[Title/Abstract])) OR (Risk Factor Score[Title/Abstract])) OR (Score, Risk Factor[Title/Abstract])) OR (Health Correlates[Title/Abstract])) OR (Correlates, Health[Title/Abstract])) OR (Social Risk Factors[Title/Abstract])) OR (Factor, Social Risk[Title/Abstract])) OR (Factors, Social Risk[Title/Abstract])) OR (Risk Factor, Social[Title/Abstract])) OR (Risk Factors, Social[Title/Abstract])) OR (Social Risk Factor[Title/Abstract]))) AND (("Enterocolitis, Necrotizing"[Mesh]) OR ((Enterocolitis, Necrotizing[Title/Abstract]) OR (Necrotizing Enterocolitis[Title/Abstract])))) AND (("Infant, Very Low Birth Weight"[Mesh]) OR (((((((Infant, Very Low Birth Weight[Title/Abstract]) OR (Very-Low-Birth-Weight Infant[Title/Abstract])) OR (Infants, Very-Low-Birth-Weight[Title/Abstract])) OR (Infant, Very-Low-Birth-Weight[Title/Abstract])) OR (Very Low Birth Weight Infant[Title/Abstract])) OR (Very-Low-Birth-Weight Infants[Title/Abstract])) OR (Very Low Birth Weight[Title/Abstract])))


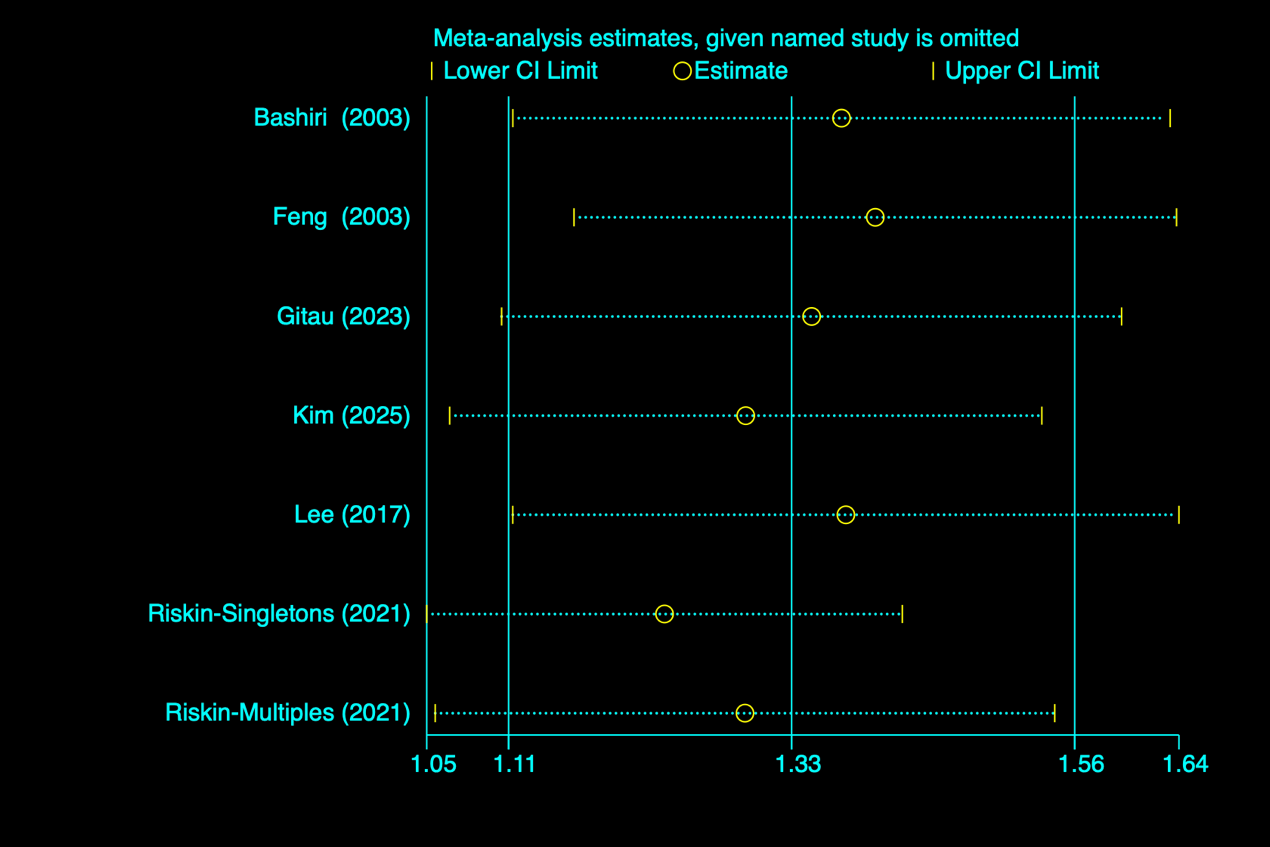


Figure S1 Sensitivity analysis of Small gestational age.


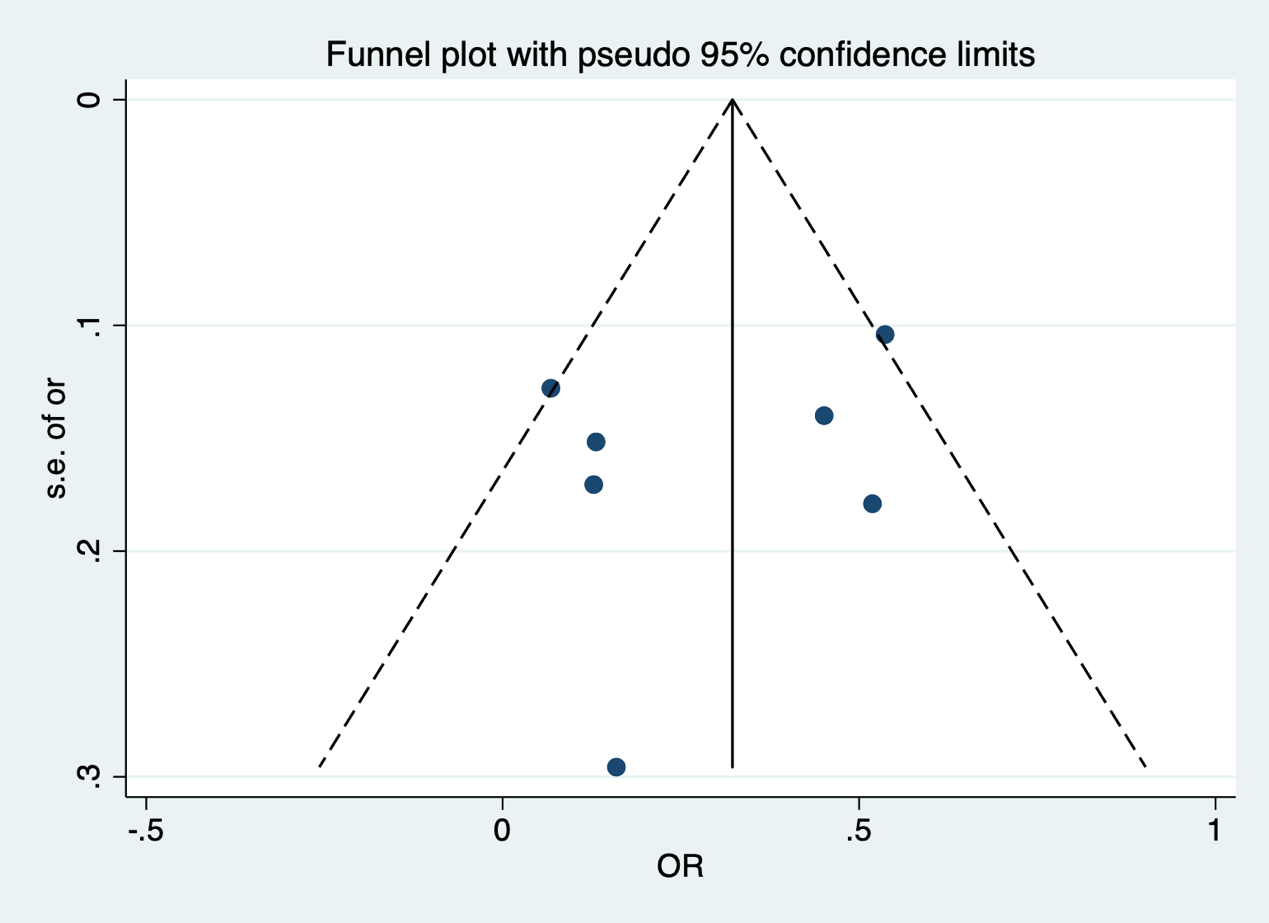


Figure S2 Funnel plot of meta-analysis for Small gestational age


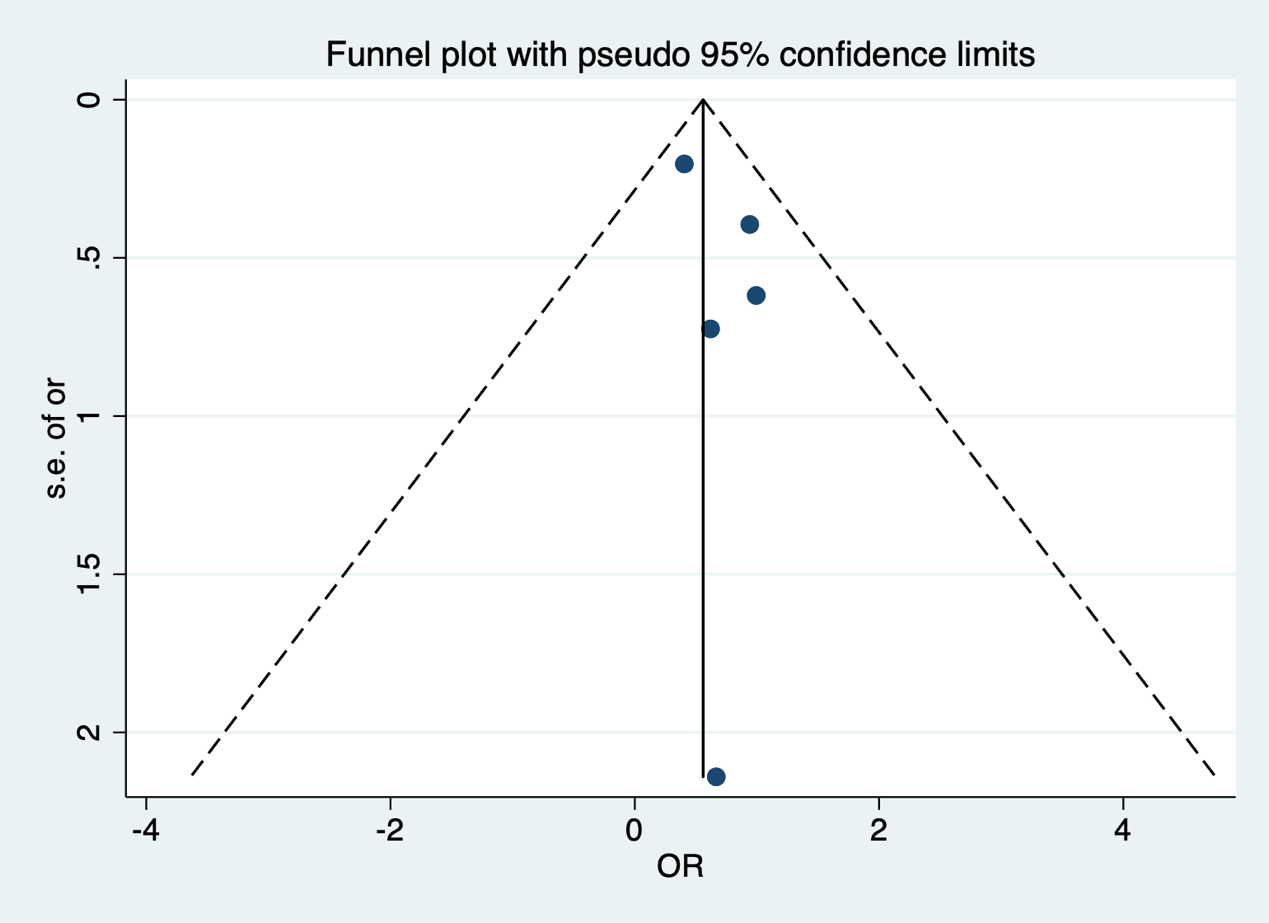


Figure S3 Funnel plot of meta-analysis for red blood cells transfusion


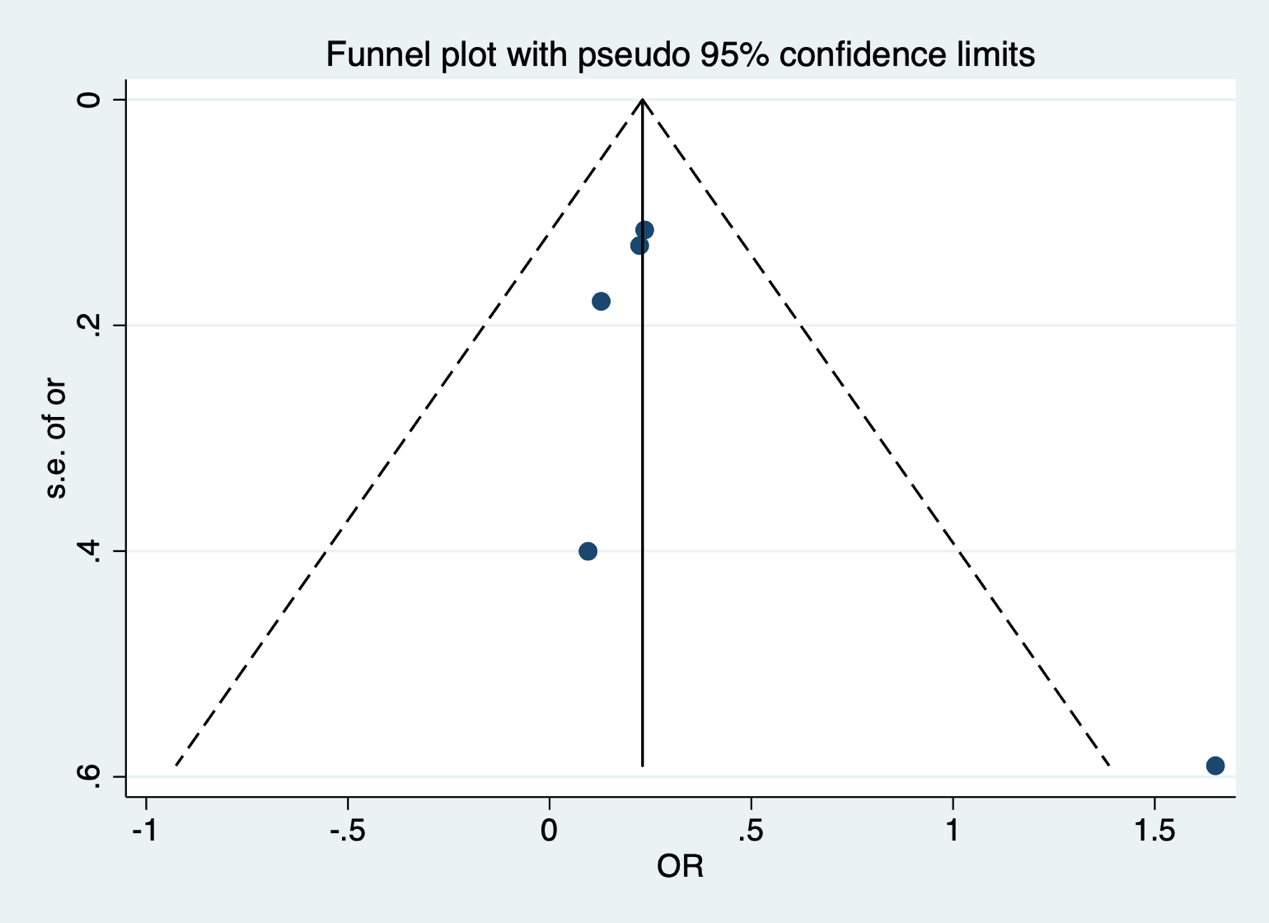


Figure S4 Funnel plot of meta-analysis for maternal hypertensive disorders


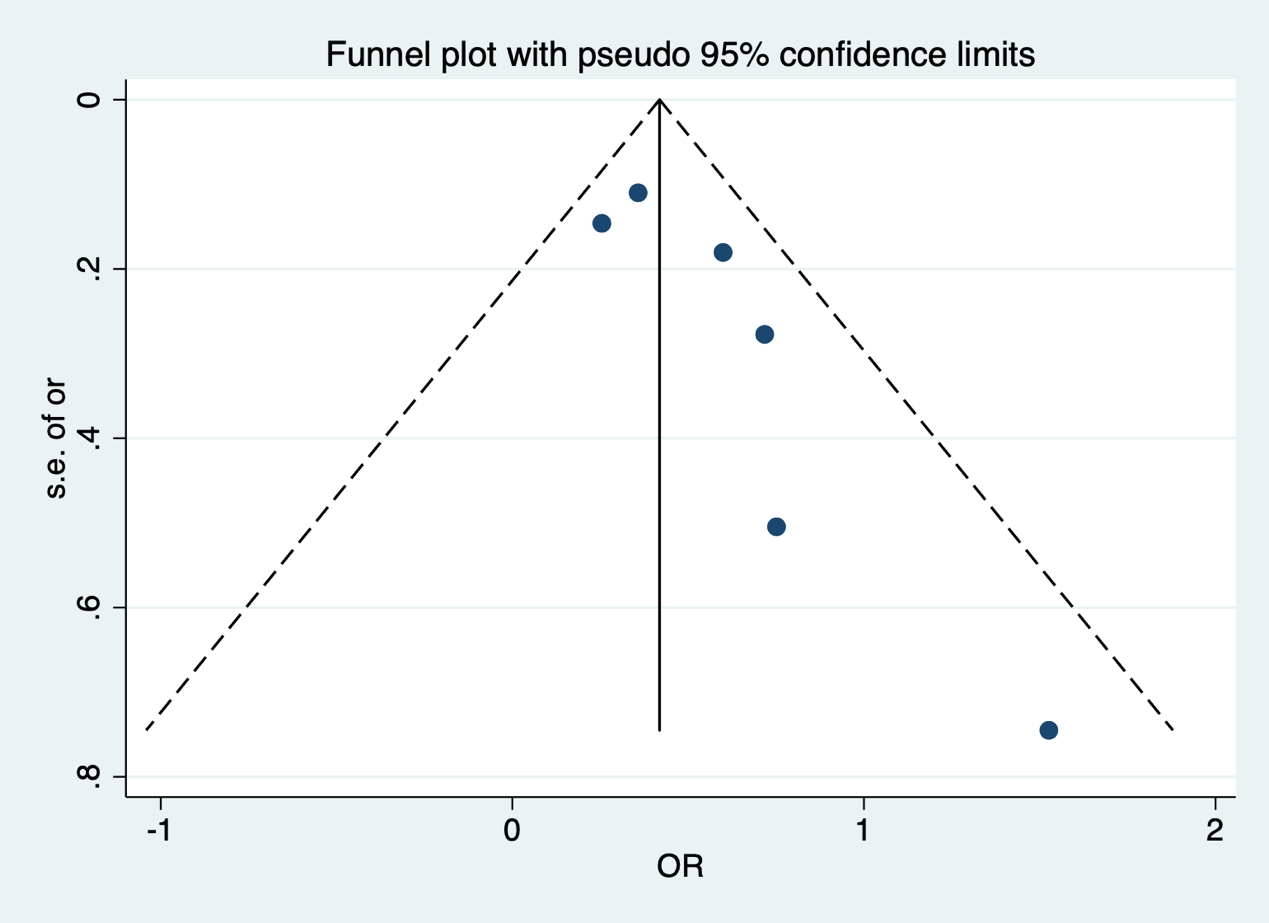


Figure S5 Funnel plot of meta-analysis for patent ductus arteriosus


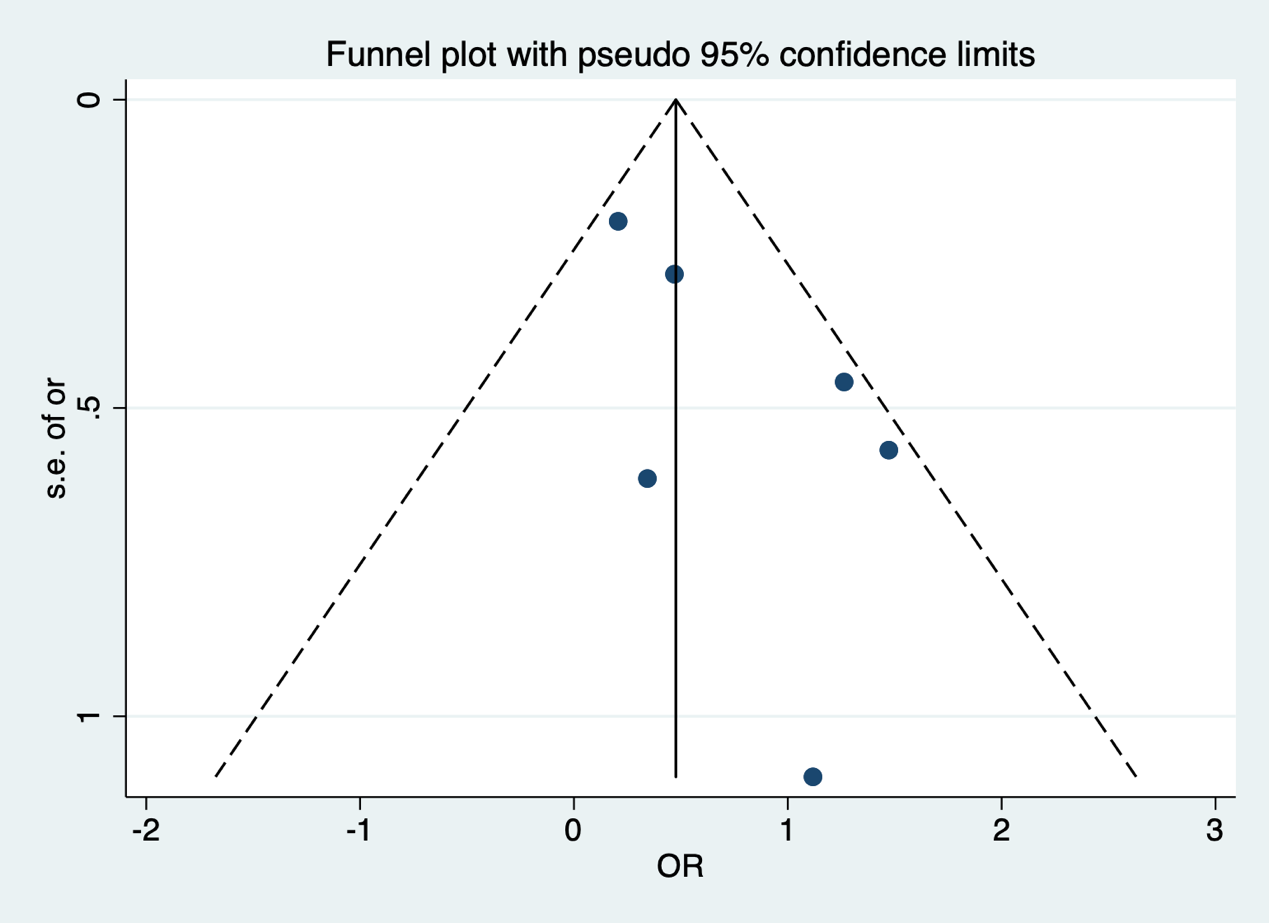


Figure S6 Funnel plot of meta-analysis for sepsis
